# A cryptic promoter in the first exon of the *SPG4 *gene directs the synthesis of the 60-kDa spastin isoform

**DOI:** 10.1186/1741-7007-6-31

**Published:** 2008-07-09

**Authors:** Giuseppe Mancuso, Elena I Rugarli

**Affiliations:** 1 Division of Biochemistry and Genetics, Istituto Neurologico 'C. Besta', Milan, Italy; 2 Department of Neuroscience and Medical Biotechnologies, University of Milano-Bicocca, Milan, Italy

## Abstract

**Background:**

Mutations in *SPG4 *cause the most common form of autosomal dominant hereditary spastic paraplegia, a neurodegenerative disease characterized by weakness and spasticity of the lower limbs due to degeneration of the corticospinal tract. *SPG4 *encodes spastin, a microtubule-severing ATPase belonging to the AAA family. Two isoforms of spastin, 68 and 60 kDa, respectively, are variably abundant in tissues, show different subcellular localizations and interact with distinct molecules. The isoforms arise through alternative initiation of translation from two AUG codons in exon 1; however, it is unclear how regulation of their expression may be achieved.

**Results:**

We present data that rule out the hypothesis that a cap-independent mechanism may be involved in the translation of the 60-kDa spastin isoform. Instead, we provide evidence for a complex transcriptional regulation of *SPG4 *that involves both a TATA-less ubiquitous promoter and a cryptic promoter in exon 1. The cryptic promoter covers the 5'-UTR and overlaps with the coding region of the gene. By using promoter-less constructs in various experimental settings, we found that the cryptic promoter is active in HeLa, HEK293 and motoneuronal NSC34 cells but not in SH-SY-5Y neuroblastoma cells. We showed that the cryptic promoter directs the synthesis of a *SPG4 *transcript that contains a shorter 5'-UTR and translates the 60-kDa spastin isoform selectively. Two polymorphisms (S44L and P45Q), leading to an early onset severe form of hereditary spastic paraplegia when present in heterozygosity with a mutant allele, fall a few nucleotides downstream of the novel transcriptional start site, opening up the possibility that they may exert their modifier effect at the transcriptional level. We provide evidence that at least one of them decreases the activity of the cryptic promoter in luciferase assays.

**Conclusion:**

We identified a cryptic promoter in exon 1 of the *SPG4 *gene that selectively drives the expression of the 60-kDa spastin isoform in a tissue-regulated manner. These data may have implications for the understanding of the biology of spastin and the pathogenic basis of hereditary spastic paraplegia.

## Background

Hereditary spastic paraplegia (HSP) is a genetically heterogeneous disorder characterized by progressive weakness and spasticity of the lower limbs owing to retrograde degeneration of the corticospinal axons [1]. *SPG4*, the gene most commonly involved in autosomal dominant HSP, encodes spastin, an ATPase belonging to the AAA family [2]. Spastin acts as a microtubule-severing protein, suggesting that axonal degeneration in HSP may depend on defective regulation of cytoskeleton dynamics in long axonal tracts [3-5]. The identification of several spastin molecular interactors involved in cell trafficking led to the proposal that the microtubule-severing activity of spastin may be coupled to specific processes and therefore, occur in a regulated manner [6].

Spastin has a complex subcellular localization. It is enriched in the centrosome in interphase and during mitosis, similarly to p60 katanin, another microtubule-severing protein [7,8]. Low levels of spastin are present in the nucleus of proliferating cells, while neurons show a prevalent cytoplasmic localization [7,9,10]. We previously found that one mechanism to regulate targeting of spastin to specific cell compartments is the alternative initiation of translation from two AUGs present in exon 1 of the *SPG4 *gene [11]. Both spastin isoforms contain a nuclear localization signal, however, the long 68-kDa spastin isoform also bears a nuclear export signal and is efficiently exported to the cytoplasm in an exportin-dependent fashion. Conversely, the shorter 60-kDa spastin isoform localizes to both the nucleus and cytoplasm upon over-expression in eukaryotic cells.

Although both spastin isoforms efficiently sever microtubules [4,5], they display several functional differences. First, the shorter isoform is the most abundant in all tissues examined, while the longer form is efficiently detectable only in brain and spinal cord [11,12]. Second, two proteins, atlastin and NA14, have been shown to interact specifically to the N-terminal region of spastin present in the long isoform but absent in the short isoform [7,13,14]. Since atlastin is in turn implicated in HSP, this observation may be of direct relevance to the pathogenesis of the disease. Third, two polymorphisms (S44L and P45Q) acting as phenotype modifiers have been identified in the long-isoform-specific region. Patients carrying a mutated allele of spastin and one of these two polymorphisms on the other allele are affected by a severe disease with an early age of onset [15-17]. Furthermore, a family has been described in which one patient with a late onset mild spastic paraplegia was homozygous for the S44L polymorphism [18].

**Figure 1:**
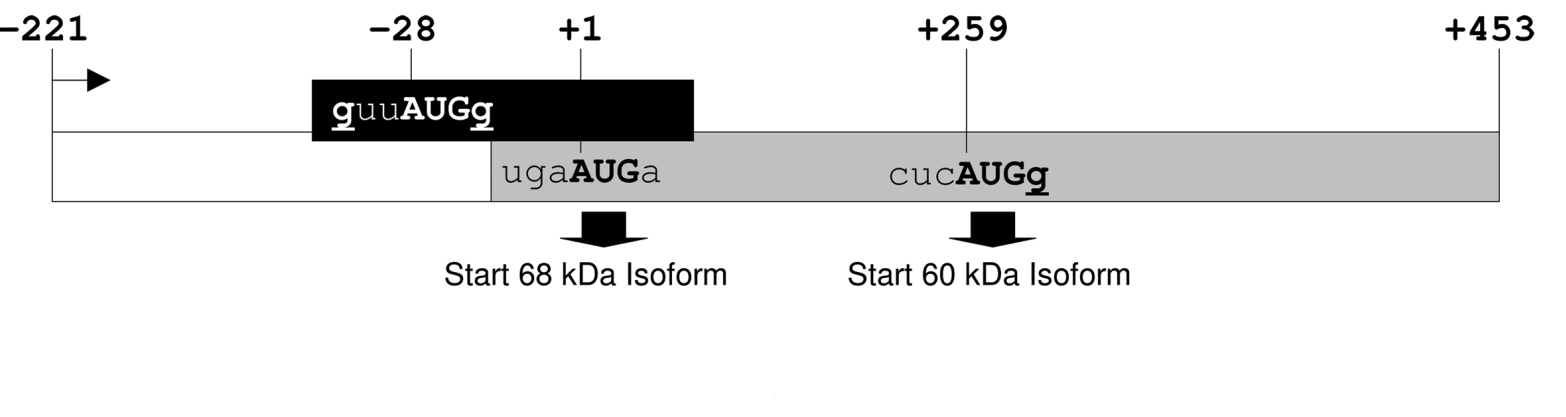
**Schematic representation of the *SPG4 *first exon**. Translation of spastin initiates from two in-frame start codons (+1 and +259). A uORF overlaps with the first in-frame AUG and may serve to divert some ribosomes to the downstream start site.

In vertebrates, initiator codons are recognized most efficiently within the context GCCRCCaugG, with the purine (R) in position -3 and the G in position +4 making the strongest contributions [19]. The main *SPG4 *open reading frame (ORF) starts with an AUG that resides in a context that deviates significantly from the consensus motif (TGAaugA). Moreover, an upstream ORF (uORF) overlaps with the main *SPG4 *ORF and contains an AUG in a good Kozak's consensus sequence (GTTaugG). It is expected that this uORF would drastically inhibit translation from the first *SPG4 *AUG, while allowing re-initiation at the second AUG that has a G in position +4 and is located at a sufficient distance from the stop codon of the uORF (Figure 1). uORFs have been recognized in genes with regulatory function and may offer a mechanism to restrict expression of a toxic product [19,20]. In some cases, limited access to the main ORF might be achieved by leaky scanning. In summary, the presence of a uORF that overlaps with the main *SPG4 *ORF, as well as the sequence context surrounding the first two AUGs in the main ORF, may explain why translation of the long spastin isoform is strongly unfavored *in vivo*. Consistent with this model, expression of an *SPG4 *cDNA containing the 5'-UTR abundantly produces the shorter protein isoform and only a limited amount of the long 68-kDa isoform [11]. Moreover, *in vitro *transcription-translation assays using a cDNA construct that starts from the first AUG supported translation of both spastin isoforms, likely reflecting leaky scanning from the first AUG [11].

Although the scanning mechanism for initiation of translation can satisfactorily explain our previous data, there might still be the possibility that translation of the short abundant 60-kDa spastin isoform occurs via direct entry of the ribosomes at the downstream AUG codon. Albeit this mechanism is well documented for certain viral genes, it is still quite controversial as to whether it occurs in mammalian genes [21,22].

While testing for the presence of an internal ribosome entry site (IRES) in the *SPG4 *mRNA, we found evidence for a cryptic promoter in exon 1, responsible for the production of a shorter mRNA specific for the 60-kDa spastin isoform. This promoter shows some degree of tissue-specificity, providing a way to regulate the production of the different spastin isoforms.

## Results

### Translation of the 60-kDa spastin isoform does not depend on an IRES

**Figure 2:**
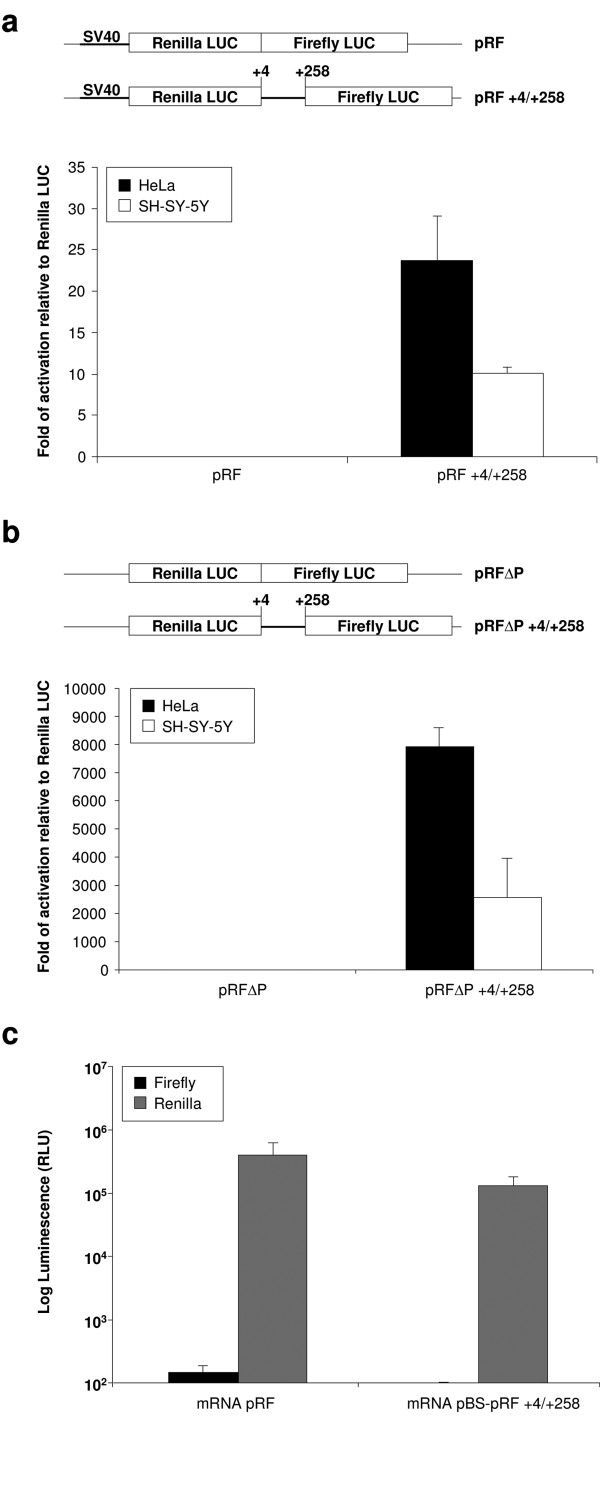
**Experiments with dicistronic vectors reveal a cryptic promoter**. (a) The sequence under analysis was cloned in a dicistronic vector between two different luciferases from *Renilla *and firefly. HeLa and SH-SY-5Y cells were transfected with the indicated constructs. Cell lysates were prepared 24 hours post-transfection and the activity of the firefly luciferase was normalized to that of the *Renilla *luciferase. For each construct at least three independent experiments were performed. (b) The same sequence was cloned into a vector lacking the SV40 promoter (pRFΔP). Cell lysates were prepared 24 hours post-transfection and the activity of the firefly luciferase was normalized to that of the *Renilla *luciferase. For each construct at least three independent experiments were performed. (c) *In vitro *transcribed dicistronic mRNAs were synthesized from the indicated linearized constructs. HeLa cells were transfected with the capped dicistronic mRNAs and *Renilla *and firefly activities were measured 8 hours after transfection. Error bars represent standard error of the mean.

Translation of the 60-kDa spastin isoform from the AUG in position 259–261 may depend on the migration of the translational machinery until it meets this AUG codon that lies in a better Kozak's sequence context than the first AUG (Figure 1). However, the program UTRScan predicts a secondary RNA structure compatible with the presence of an IRES, immediately upstream of the second AUG, suggesting that the short spastin isoform could be synthesized through a cap-independent mechanism. To test this possibility, we cloned the *SPG4 *cDNA sequence between the first and second ATG into a widely used dicistronic vector, pRF (construct pRF +4/+258). This vector contains the SV40 promoter directing the expression of a dicistronic RNA encoding the *Renilla *luciferase as the first cistron and the firefly luciferase as the second. This plasmid was transfected in HeLa and SH-SY-5Y neuroblastoma cell lines and the *Renilla *and firefly luciferase activities were measured. The construct pRF +4/+258 displayed a high firefly activity in HeLa cells compared with the control empty vector (Figure 2a), consistent with the possibility that the region between the two AUGs in the first exon of *SPG4 *might contain a functional IRES. This construct was less active in SH-SY-5Y cells.

Although the dicistronic test has been considered the gold standard for testing the existence of functional IRES elements, a major drawback of this approach is that it cannot distinguish between IRES activity and the presence of a cryptic promoter [23]. To exclude this possibility, we cloned the same *SPG4 *sequence into a promoter-less pRF vector (pRFΔP), in which the SV40 promoter has been removed (Figure 2b). Both *Renilla *and firefly luciferase activities were almost undetectable when the empty pRFΔP vector was transfected, whereas a dramatic increase of firefly activity was observed for the pRFΔP +4/+258 construct in HeLa cells, strongly suggesting the presence of a promoter activity in the first exon of *SPG4 *(Figure 2b). Again, the fold of activation was lower in SH-SY-5Y cells (Figure 2b).

The presence of a strong promoter in the region under analysis could mask the presence of the IRES, hampering the detection of its functionality. To circumvent this problem, an effective method is direct transfection of the dicistronic RNAs [23]. To this end, *in vitro*-transcribed capped dicistronic mRNAs were transfected into HeLa cells and the activities of both *Renilla *and firefly luciferases were measured. The firefly activities of both the empty vector and the pRF +4/+258 were barely detectable, while the *Renilla *luciferase activities were comparable, indicating that the first exon of *SPG4 *does not contain an IRES element (Figure 2c).

### A minimal ubiquitous *SPG4 *promoter

**Figure 3:**
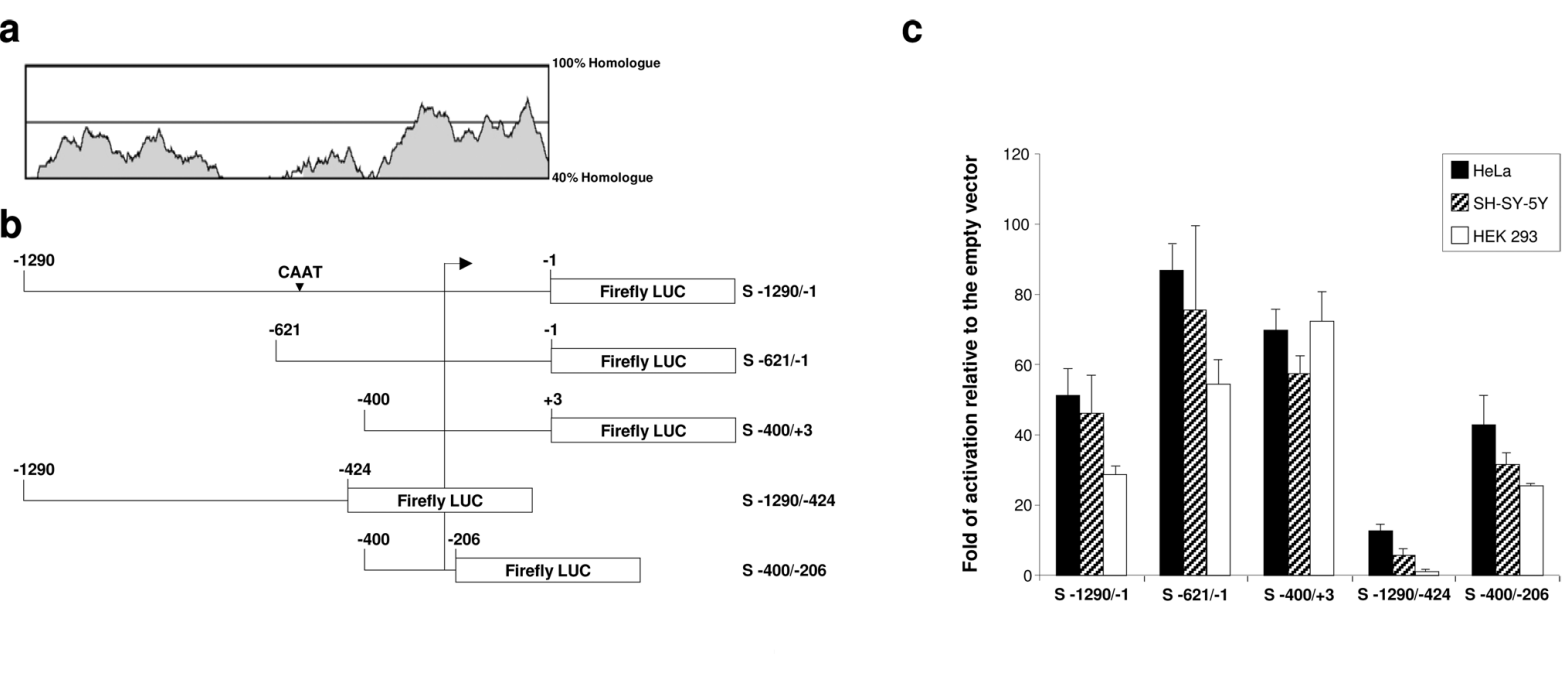
**Analysis of the *SPG4 *minimal promoter**. (a) Sequence comparison using mVISTA between human and mouse genomic regions upstream of the first ATG of *SPG4 *demonstrates extensive sequence conservation. (b) Schematic representation of the constructs used. Different genomic sequences were cloned upstream of the firefly luciferase reporter gene. The arrow indicates the transcriptional start sites of the reference *SPG4 *sequence. The position of a putative CAAT box is shown. (c) HeLa, SH-SY-5Y and HEK293 cells were cotransfected with the indicated constructs and with a CMV-*Renilla *luciferase plasmid. Cell lysates were prepared 24 hours post-transfection and the activity of the firefly luciferase was normalized to that of *Renilla *luciferase. For each construct at least three independent experiments were performed using different DNA preparations. Error bars represent standard error of the mean.

The finding of promoter activity in the region between the two ATGs prompted us to study the regulatory sequences of the *SPG4 *gene. Bioinformatic analysis of the genomic region upstream of the transcriptional start site (defined as in the reference sequence AB029006) does not identify any TATA box, but detects several CG boxes and a CAAT box in position -597. Furthermore, sequence comparison between the human and mouse *SPG4 *genomic sequence shows a high degree of sequence conservation in the 5'-UTR of *SPG4 *and in a region of 400 base pairs (bp) upstream of the putative initiation of transcription, suggesting that this region may contain important regulatory elements (Figure 3a).

To define the minimal genomic region that confers basic expression of the *SPG4 *gene, we tested the ability of different fragments of the genomic region upstream of the first ATG of the *SPG4 *gene to drive the expression of the luciferase gene in transiently transfected HeLa, HEK293 and SH-SY-5Y cells (Figure 3b). The activities of these promoters were measured by a luciferase assay and considered as fold of induction in respect to the activity of the empty vector. We did not find any cell-specific difference in the activities of the different fragments in the three cell lines (Figure 3c). The construct that showed higher promoter activity was S -621/-1, which contained the highly conserved, 400 bp-genomic region and the 5'-UTR. Inclusion of an additional 669 bp upstream of this region led to a certain decrease of promoter activity, while removal of a sequence of approximately 220 bp containing the CAAT box (S -400/+3) did not reduce significantly the promoter activity. Deletion of the 5'-UTR and approximately 200 bp upstream of the transcription initiation site (construct S -1290/-424) completely abolished promoter activity, while the removal of only the 5'-UTR (S -400/-206) reduced the basal transcriptional activity (Figure 3c). These experiments identified a region of 400 bp upstream of the first ATG as a minimal promoter region active in all cell lines tested and point to a role of the 5'-UTR to sustain basic ubiquitous *SPG4 *expression.

### A tissue-specific cryptic promoter in the first exon of *SPG4*

**Figure 4:**
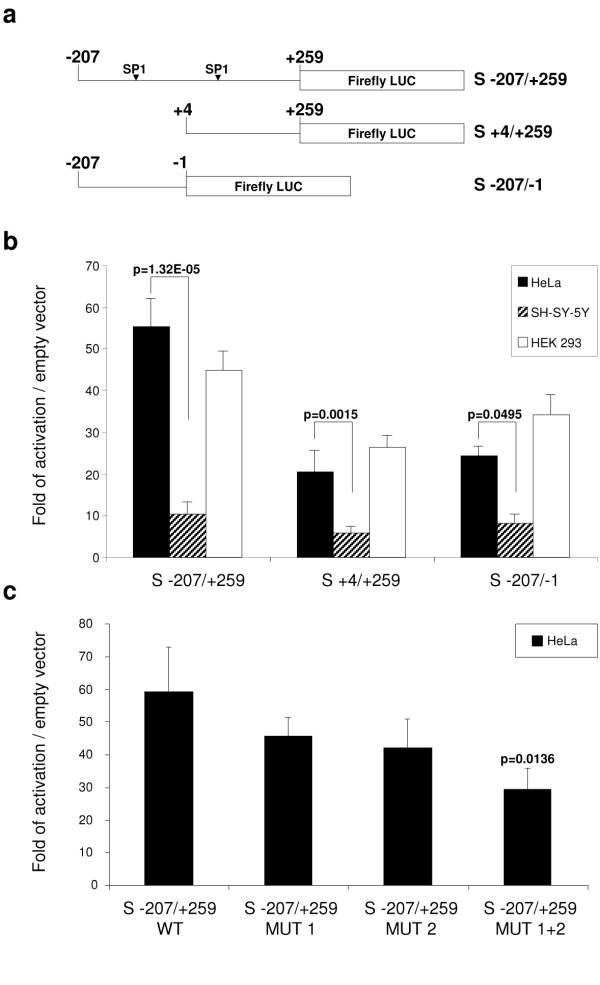
**Identification of a cryptic promoter in *SPG4 *exon 1**. (a) Schematic representations of the firefly luciferase reporter constructs used. The position of the predicted *Sp1 *sites is indicated. (b) HeLa, SH-SY-5Y and HEK293 cells were cotransfected with the indicated constructs and with a CMV-*Renilla *luciferase plasmid. Cell lysates were prepared 24 hours post-transfection and the activity of the firefly luciferase was normalized to that of *Renilla *luciferase. For each construct at least three independent experiments were performed using different DNA preparations. (c) Mutation of each and both predicted *Sp1 *sites were generated in the S -207/+259 construct and tested in HeLa cells as described above (*n *= 3). Error bars represent standard error of the mean. The *P*-value of Student's *t *test is shown.

The promoter activity observed in the region within the two ATGs in the experiments with the promoter-less pRFΔP vector, as well as the role of the 5'-UTR for basal expression, induced us to examine in detail the potential presence of regulatory sequences in exon 1. We cloned different regions of the first exon of *SPG4 *upstream of the firefly luciferase gene and tested their promoter activity in all cell lines (Figure 4a). We found a strong promoter activity in both HeLa and HEK293 cells in the region that starts immediately downstream of the putative transcriptional start site (TSS) and include both ATGs (S -207/+259) (Figure 4b). This activity, although decreased, is still present in constructs that contain only the coding region (S +4/+259) or the 5'-UTR (S -207/-1). We define the whole region between the canonical TSS, as defined in public databases, and the AUG in position 259–261, as a cryptic promoter. Notably, the activity of this cryptic promoter appears to display some degree of cell-line specificity, being highly functional in HeLa cells and HEK293 and significantly less in SH-SY-5Y cells (Figure 4b), thus confirming our previous observations with the promoter-less dicistronic vector.

We used the TRANSFAC program to identify binding sites for known transcription factors in the cryptic promoter region. This allowed us to identify two putative *Sp1 *binding sites that were conserved in the human and mouse genomes (Figure 4a). Site-directed mutagenesis was employed to insert mutations into the upstream, the downstream, or both *Sp1 *sites in the construct S -207/+259. Transfection of the mutated constructs showed a significant reduction of the cryptic promoter activity in HeLa cells only when both *Sp1 *sites are mutagenized (Figure 4c).

**Figure 5:**
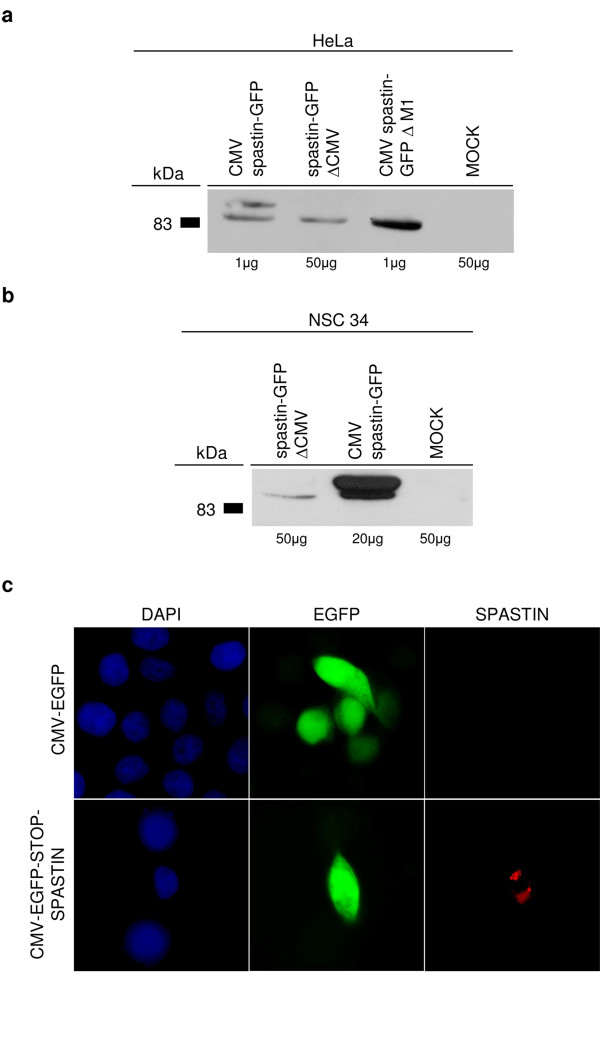
**The cryptic promoter mediates expression of the short spastin isoform *in vivo***. (a) HeLa cells were transfected with a CMV-spastin-GFP, a CMV-spastin-ΔM1 or a spastin-GFP- ΔCMV construct. Cell lysates were prepared 48 hours post-transfection and subjected to sodium dodecyl sulfate-polyacrylamide gel electrophoresis. Immunoblotting for transfected spastin was performed with the S51 polyclonal antibody. The CMV-spastin-GFP plasmid drives expression of two spastin isoforms starting from the first and second methionine, as previously described [11]. Consistently, the CMV-spastin- ΔM1 construct produces only the shorter isoform. Notably, the promoter-less spastin construct synthesizes the short isoform, albeit at lower level, indicating that the cryptic promoter of spastin is active *in vivo*. Below each lane, the amount of transfected cell lysate loaded is indicated. (b) Similar results were obtained in the murine immortalized motoneuronal cell line NSC34. (c) The empty CMV-EGFP vector and CMV-EGFP-STOP-spastin construct were transfected in HeLa cells. Immunofluorescence was performed 48 hours after transfection. Transfected cells were detected by enhanced green fluorescent protein epifluorescence, while spastin was revealed using the S51 polyclonal antibody. Cells expressing high levels of GFP also synthesize low levels of spastin. Note the different pattern of GFP (diffuse) and spastin staining (discrete, as described previously [11]).

We previously showed that when spastin is expressed in mammalian cells, two isoforms are produced, starting from the first and second methionine [11]. The amount of the shorter isoform increases when the 5'-UTR is included in the construct [11]. *In vitro *transcription-translation experiments suggested that this is largely due to alternative initiation of translation [11]. However, our novel findings suggest that transcriptional regulation could contribute to the production of the shorter isoform through the use of the cryptic promoter. To test this possibility, we removed the CMV promoter from a CMV-spastin-GFP construct and analyzed the ability of the cryptic promoter (in this construct represented only by the region +4/+259) to drive the expression of the short spastin isoform after transfection in different cell lines. We found that a short spastin-GFP isoform, with a size consistent with initiation of translation at the second AUG, is produced in this condition in Hela, HEK293 and murine spinal motoneuronal NSC34 cells (Figure 5a and 5b and data not shown). To confirm this data, we generated a construct containing the GFP reporter under the control of the CMV promoter followed by a stop codon and by the coding region of spastin (CMV-EGFP-STOP-Spastin). Such a construct could express spastin only if the region between the first two ATGs functions as a promoter. Consistently, transfected cells with high levels of GFP expression showed low levels of spastin expression, detected with a specific antibody (Figure 5c).

In conclusion, both reporter and expression studies with promoter-less constructs strongly indicate that the first *SPG4 *exon contains a cryptic promoter that may contribute to produce the 60-kDa isoform in several cell types *in vivo*.

### Two phenotype-modifier polymorphisms lie within the cryptic promoter

**Figure 6:**
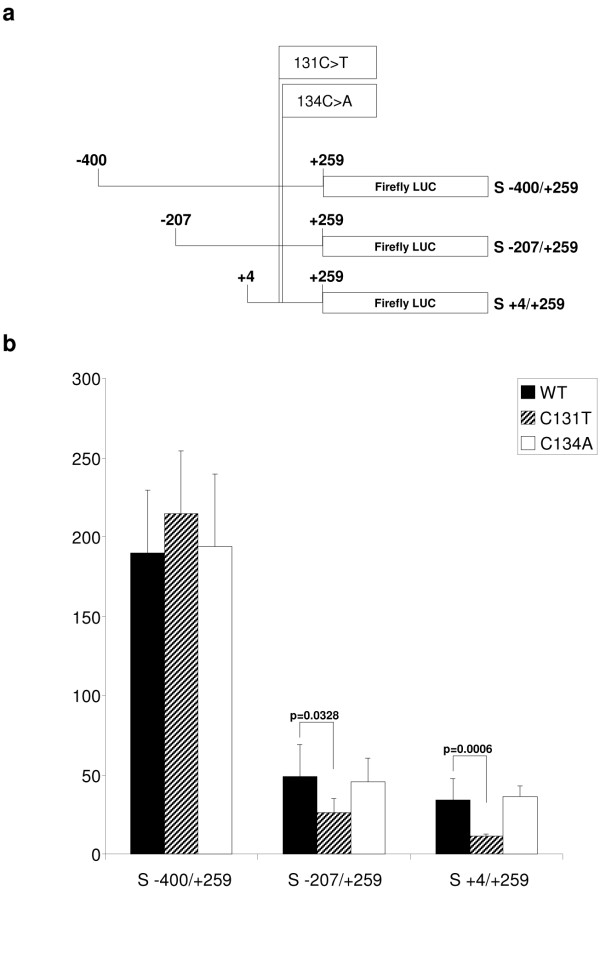
**The role of c.131C>T and c.134C>A polymorphisms on cryptic promoter activity**. (a) Schematic representations of the firefly luciferase reporter constructs used. The position of both polymorphisms is indicated. (b) HeLa cells were transfected with the indicated constructs together with a plasmid containing CMV-*Renilla *luciferase. Cell lysates were prepared 24 hours post-transfection and the activity of the firefly luciferase was normalized to that of *Renilla *luciferase. For each construct at least three independent experiments were performed using different DNA preparations. Error bars represent standard error of the mean. The *P*-value of Student's *t *test is shown.

The S44L and P45Q (c.131C>T and c.134C>A) polymorphisms in the *SPG4 *gene act as phenotype-modifiers. Patients that bear one of these polymorphisms and a canonical *SPG4 *mutation on the other allele show an early age onset of HSP and rapid progression of symptoms [15,16]. Since these nucleotide changes fall into the newly identified cryptic promoter, we tested their capability to affect the promoter activity. The polymorphisms were inserted by mutagenesis in the constructs S -207/+259 and S +4/+259 (Figure 6a). The activity of these mutagenized promoters was tested in HeLa cells and compared with wild-type constructs. The presence of the c.131C>T substitution significantly diminished the activity of the promoter by about a half, while no effect was detected with the c.134C>A substitution (Figure 6b). When the substitutions were inserted in the context of a larger promoter, also containing part of the ubiquitous minimal promoter (S -400/+259), no change in activity was observed for either (Figure 6b).

### Identification of an endogenous *SPG4 *transcript specific for the short spastin isoform

The previous experiments strongly suggest the existence of a cryptic promoter in the first exon of the *SPG4 *gene. Differentially regulated, alternative TSSs are a common feature in protein-coding genes and commonly generate alternative N-termini [24]. Genome-wide analyses, using short tags derived from the 5'-ends of capped RNAs (CAGE), oligocapping methods and full-length cDNA collections, can be publicly accessed in the CAGE analysis website and in the database of transcriptional start sites (DBTSS). We searched these databases for TSSs within the human *SPG4 *gene. Remarkably, we found that both databases identify an alternative promoter located within exon 1, downstream of the first ATG, defined by a clustering of TSSs separated by fewer than 500 bp. The tags derive from HEK293 cells, as well as from different tissues, including brain tissue. A summary of these data is represented in Additional file 1 and additional file 2.

**Figure 7:**
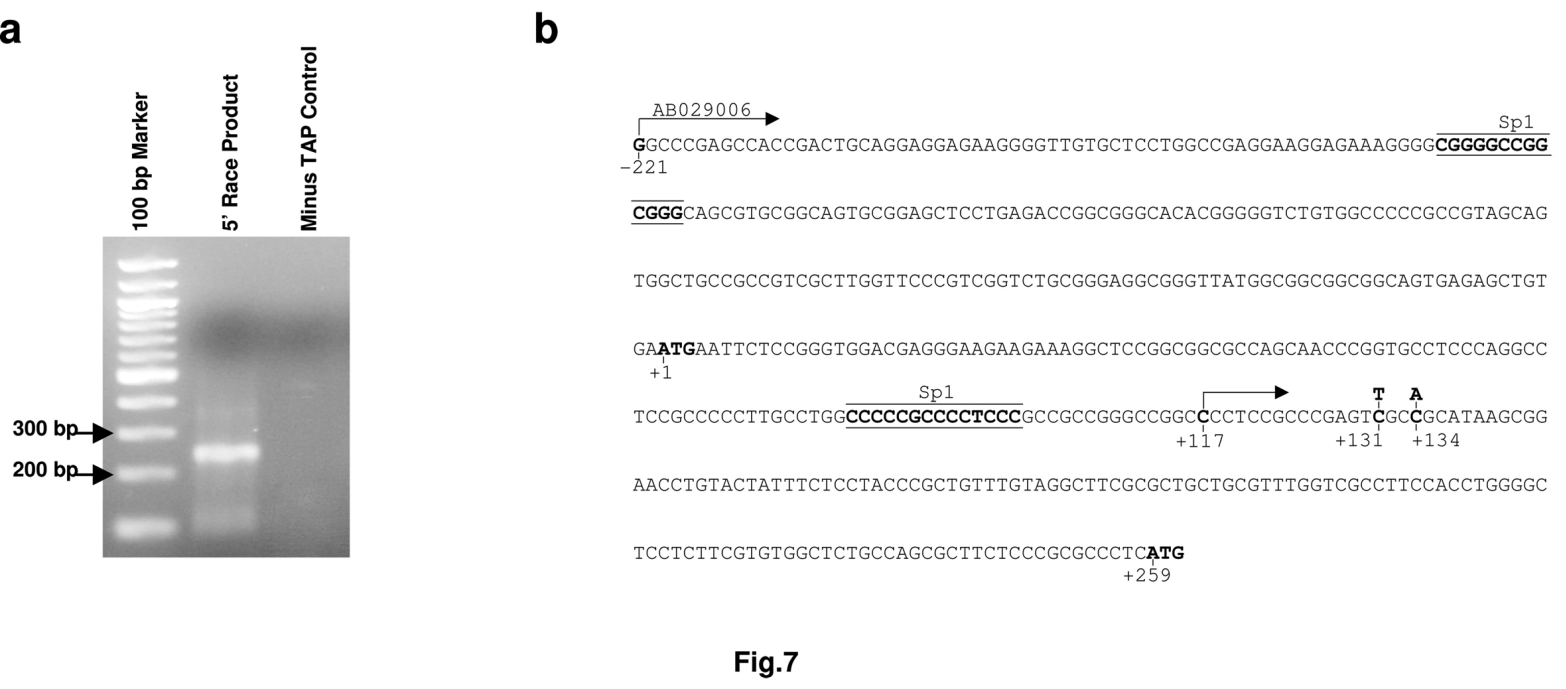
**An endogenous *SPG4 *transcript specific for the 60-kDa spastin isoform**. (a) 5'-end RACE performed on total RNA extracted from HeLa cells detects a specific product of about 250 base pairs that is lacking in the minus tobacco acid pyrophosphatase control sample. (b) Sequence of the *SPG4 *first exon starting from the transcriptional start site as defined in public databases and ending with the second ATG in position 259–261. *Sp1 *sites are underlined, the position of the c.131C>T and c.134C>A polymorphisms and the two in-frame ATGs are shown in bold, while arrows indicate the traditional and novel transcriptional start site found in this study.

To gain further experimental proof that the cryptic promoter is responsible for the synthesis of a short *SPG4 *mRNA, we performed 5'-end RACE experiments in both HeLa and SH-SY-5Y cells. Total RNA was isolated from the cells. Truncated or uncapped RNA molecules were removed by a phosphatase treatment. Subsequently, the caps were eliminated by treatment with tobacco acid pyrophosphatase (TAP), and an adapter oligonucleotide was ligated to the 5'-ends. After nested amplification with gene-specific primers located downstream of the second AUG, we could amplify in HeLa cells a specific product of about 250 bp, which was absent from the minus TAP control reaction (Figure 7a). This product was cloned and sequenced and found to initiate from nucleotide +117. This transcript therefore, contains an ORF that starts with AUG 259–261 and encodes the short 60-kDa spastin isoform. Both *Sp1 *sites are located upstream of the beginning of the novel transcript, while the polymorphisms c.131C>T and c.134C>A appear to be positioned a few bases downstream (Figure 7b). Notably, this TSS corresponds to two tags identified in HEK293 in the DBTSS database. We could not obtain a similar 5'-end RACE product in SH-SY-5Y cells, consistent with the lower activity of the cryptic promoter in this cell line (not shown).

## Discussion

Haplo-insufficiency of spastin causes HSP, suggesting that tight control of the protein levels is required for axonal integrity. We previously showed that the *SPG4 *gene synthesizes two isoforms of spastin (68 kDa and 60 kDa, respectively), depending on the alternative initiation of translation from two AUGs in the first exon [11]. Regulation of the expression of protein isoforms simply based on inefficient translation or leaky scanning is, however, hard to achieve. Here, we report a transcriptional mechanism of *SPG4 *regulation that may contribute to the production of a different ratio of long and short spastin isoforms in tissues.

We identified a ubiquitous spastin minimal promoter and found evidence for a tissue-specific cryptic promoter in the first exon of the gene. An evolutionary highly conserved region of 400 bp upstream of the first in-frame AUG of the *SPG4 *gene was sufficient to provide basal expression in HeLa, HEK293 and SH-SY-5Y cells. This region does not contain a TATA box, but includes several *cis*-acting, GC-rich elements, suggesting that the *SPG4 *promoter belongs to the vast category of TATA-less promoters common to mammalian housekeeping genes [25]. Inclusion in the reporter constructs of upstream genomic regions did not significantly increase transcriptional activity. Furthermore, deletion of a putative CAAT box, not conserved in the mouse, did not decrease substantially promoter activity. In contrast, a certain drop in activity was found when the majority of the 5'-UTR of the gene was removed from all the constructs tested. A possible explanation is that the 5'-UTR itself may contain additional TSS or regulatory elements that cooperate with upstream sequences to allow basal transcription of the *SPG4 *gene. Consistently high levels of sequence conservation are observed in the 5'-UTR among different species from human to chicken.

The latter hypothesis is supported by the finding of a cryptic promoter in the first exon of the *SPG4 *gene. The region between the most upstream TSS (corresponding to position -221) and the first ATG, and the region between the first and the second in-frame ATGs, both, alone and even stronger in combination, are able to drive the expression of a reporter gene in promoter-less vectors. Collectively, we define these regions in *SPG4 *exon 1 as a cryptic promoter. Furthermore, promoter-less constructs containing only the coding sequence of spastin drove the expression of the shorter spastin isoform in Hela, HEK293 and NSC34 cells. These are murine immortalized spinal motoneurons that express both long and short spastin isoforms [11]. This result suggests that the cryptic promoter may also be active in neurons implicated in human pathology. Remarkably, the cryptic promoter shows some degree of tissue-specificity, as shown by low activity in the neuroblastoma-derived SH-SY-5Y cells.

The presence of shorter capped *SPG4 *mRNAs is supported by the successful identification of a novel *SPG4 *transcript that starts downstream of the first AUG in HeLa cells by 5'-end RACE experiments. Moreover, our experimental data are consistent with high-throughput, genome-wide studies, which identified a cluster of TSSs within both the human and murine *SPG4 *genes located in close proximity to the TSS of the novel transcript identified in our study. The previous results strongly suggest that the *SPG4 *gene has multiple core promoters containing multiple TSSs, the use of which generates diversity, not only in the transcripts, but most importantly, in the proteins produced. A similar scenario is emerging with more and more frequency from studies of mammalian core promoters [25]. As expected for a broad promoter with multiple TSSs, several CpG islands boxes and multiple binding sites for the transcription factor *Sp1 *are present in the cryptic promoter. It has been suggested that *Sp1 *may direct the basal machinery to form a pre-initiation complex within a loosely defined window [26]. Mutagenesis of two evolutionary conserved *Sp1 *sites decreased the activity of the cryptic promoter, suggesting that *Sp1 *or *Sp *family member transcription factors may bind to the cryptic promoter. *Sp1 *elements are required for the expression of many ubiquitous, tissue-specific and viral genes [27]. Interestingly, *Sp1 *levels decrease with cellular aging [28]. Further studies however, are required to define the transcription factors involved in *SPG4 *expression.

Western blot analysis strongly indicates that the 60-kDa spastin isoform is predominant in many tissues and cells [11,12]. Based on our data, we propose that a combination of transcriptional and translational mechanisms is employed in concert to modulate the levels of spastin isoforms in cells. At the transcriptional level, cells may synthesize the 60-kDa isoform simply through the production of a shorter transcript that possesses as first in-frame AUG, the one in position 259–261. However, an additional mechanism to ensure preferential synthesis of the 60-kDa spastin isoform likely arises during translation, due to several constraints imposed on translation from the first in-frame AUG, such as the presence of a 73% GC-rich 5'-UTR, an overlapping uORF and a poor Kozak's context [19]. Indeed, in our experiments with spastin expression constructs, it is clear that the short spastin isoform is expressed at high levels when the *SPG4 *coding sequence is under the control of the CMV promoter, suggesting that translation of this isoform occurs even when the synthesis of a longer mRNA is favored.

Our study tends to exclude a role for a cap-independent mechanism through recognition of an IRES in the translation of the spastin 60-kDa short isoform. This latter mechanism has been extensively demonstrated in viral transcripts, and more recently has also been found in a number of eukaryotic transcripts, whose translation needs to occur also in circumstances in which cap-dependent translation is inhibited. Functional IRES elements have been proposed in several eukaryotic genes, but subsequent studies using more sensitive procedures have questioned the validity of several of them [29-31]. Similarly, we showed by direct RNA transfection of a dicistronic transcript that the predicted IRES in the *SPG4 *exon 1 is not functional, further confirming the imprecision of bioinformatic approaches to predict IRES sequences and stressing the importance of adequate functional validation.

It remains to be established why several mechanisms have evolved to maintain the low levels of the long 68-kDa spastin isoform in most cells and tissues. This apparently seems to contrast with the evolutionary conservation of the first AUG and even of the uORF in several organisms, and may point to the need for a regulated expression of this isoform, or a possible toxic effect if expressed at a high level.

The identification of the cryptic *SPG4 *promoter and a shorter *SPG4 *transcript may have implications in human pathology. We found that a previously reported polymorphism (c.131C>T) that acts as a disease modifier falls into the cryptic promoter region and decreases its activity significantly. Notably, this polymorphism is a few base pairs downstream of the TSS of the novel transcript described here and therefore, within a bona fide *SPG4 *core promoter. However, a second polymorphism, c.134C>A, did not affect cryptic promoter activity, casting doubts on whether these polymorphisms actually act transcriptionally. Further studies on cell lines derived from HSP families in which both a mutant *SPG4 *allele and the polymorphism segregate are needed to address this issue.

## Conclusion

Our study describes alternative promoter usage and heterogeneity of transcription initiation for the *SPG4 *gene. A canonical promoter has features typical of housekeeping genes, while a cryptic promoter in the 5'-UTR and coding region of spastin seems to provide tissue-specificity. Use of these alternative promoters generates *SPG4 *mRNAs with 5'-UTRs of different length and with different AUGs driving the production of different spastin isoforms. Our study emphasizes the need to take into account *SPG4 *complex transcriptional regulation to achieve a better understanding of the biology of spastin and the pathogenic effect of mutations or polymorphisms located in the first exon of the gene.

## Methods

### Constructs

In all numeric references in this study, nucleotide +1 corresponds to the A of the first ATG codon according to den Dunnen and Antonarakis [32].

#### Dicistronic constructs (pRF)

The +4/+258 region of *SPG4 *was amplified by polymerase chain reaction (PCR) from human genomic DNA using *Pfu *Ultra (Stratagene) and cloned SpeI/NcoI into pRF and pRFΔP (Fw: 5'-GC**AGTACT**TAATTCTCCGGGTGGACGA-3', Rev: 5'-AT**CCATGG**GAGGGCGCGGGAGAAGCG-3', *Spe*I and *Nco*I sites in bold, respectively). Both dicistronic vectors were a kind gift from Dr J-T Zhang [30]. For RNA transfection experiments, the dicistronic cassette was excised from pRF +4/+258 using a *Bam*HI/*Nhe*I digestion and then cloned *Bam*HI/*Eco*RV into the multiple cloning site of pBluescript KS (Stratagene).

#### Spastin promoter constructs (S)

All of these constructs were obtained by cloning different portions of the human genomic sequence upstream of the *SPG4 *start codon into the pGL3 vector (Promega). The S -1290/-1 insert was amplified by PCR using Pfu Ultra (Stratagene) from human genomic DNA and cloned in the *Xho*I/*Hind*III sites of pGL3 vector (Fw: 5'-AT**CTCGAG**AACCCAGCAGCTCTGGGGGA-3', Rev: 5'-AT**AAGCTT**TCACAGCTCTCACTGCCGCC-3', *Xho*I and *Hind*III sites in bold, respectively). S -621/-1 was obtained from S -1290/-1 by *Sma*I/*Eco*RV excision and self-ligation. S -207/-1 was obtained from S -1290/-1 by *Kpn*I/*Pst*I excision and self-ligation. S -1290/-424 was obtained from S -1290/-1 by *Hind*III/*Pst*I excision and self-ligation. The S -400/+259 insert was amplified by PCR using Pfu Ultra (Stratagene) from human genomic DNA and cloned *Xho*I/*Hind*III (Fw: 5'-AT**CTCGAG**TGGGAACTGTAGTTGAGT-3', Rev: 5'-AT**AAGCTT**CGGAGCTCCTCCTGGCTG-3', *Xho*I and *Hind*III sites in bold, respectively). S -207/+259 was obtained from S -400/+259 by *Sma*I/*Pst*I excision and self-ligation. S +4/+259 was obtained from S -400/+259 by *Sma*I/*Eco*RI excision and self-ligation. S -400/+3 was obtained from S -400/+259 by *Eco*RI/*Hind*III excision and self-ligation. S -400/-206 was obtained from S -400/+259 by *Pst*I/*Hind*III excision and self-ligation.

#### Expression constructs

Spastin-GFP-ΔCMV was obtained from CMV-spastin-GFP vector [3] by digestion with *Nru*I/*Kpn*I and self-ligation. Spastin-GFP-ΔM1 was obtained from spastin-GFP vector by digestion with *Eco*RI/*Kpn*I and self-ligation. To obtain the CMV-EGFP-STOP-spastin construct, the *SPG4 *coding region was cloned blunt in the *Not*I site of pEGFP N°2 (Clontech).

### Site-directed mutagenesis

Site-directed mutagenesis was performed by PCR reactions using *Pfu *Ultra (Stratagene). After amplification, 10 U of *Dpn*I were added to the PCR product and incubated for 1 hour at 37°C. The mutagenized DNA was transformed into *E. coli XL1Blue *super-competent cells. The c.131C>T polymorphism was introduced into S -400/+259, S -207/+259 and S +4/+259 vectors using the following set of oligos: Fw: 5'-GCCCCTCCGCCCGAGT**T**GCCGCATAAGCGGAAC-3', Rev: 5'-GTTCCGCTTATGCGGC**A**ACTCGGGCGGAGGGGC-3' (mismatches reported in bold). The c.134C>A polymorphism was introduced into S -400/+259, S -207/+259 and S +4/+259 vectors using the following set of oligos: Fw: 5'-CCTCCGCCCGAGTCGC**A**GCATAAGCGGAACCTG-3', Rev: 5'-CAGGTTCCGCTTATGC**T**GCGACTCGGGCGGAGG-3' (mismatches reported in bold). Mutagenesis of *Sp1 *sites was introduced in the S-207/+259 construct. The upstream *Sp1 *site was mutated using this set of oligos: Fw: 5'-AGGAAGGAGAAAGGGG**AA**GGGC**AA**GCGGGCAGCGTGCGG-3', Rev: 5'-CCGCACGCTGCCCGC**TT**GCCC**TT**CCCCTTTCTCCTTCCT-3' (mismatches reported in bold). The downstream *Sp1 *site was mutated using the following set of oligos: Fw: 5'-CCCTTGCCTGGCCCC**AA**CCCC**AA**CCGCCGCCGGGCCGGC-3', Rev: 5'-GCCGGCCCGGCGGCGG**TT**GGGG**TT**GGGGCCAGGCAAGGG-3' (mismatches reported in bold). All mutagenized vectors were controlled by DNA sequencing.

### DNA sequencing

DNA sequencing was performed by using a 3100 Genetic Analyzer (Applied Biosystems) and BigDye Terminator v1.1 Cycle Sequencing kit (Applied Biosystems) according to the manufacturer's specifications.

### Luciferase assays

*Renilla *(RL) and firefly luciferase (FL) activities were measured using the Dual-Luciferase Reporter System (Promega) and a Victor^2 ^1420 Multilabel Counter (Perkin Elmer). At 24 hours post-transfection, 20 μl of cell lysate was combined sequentially with FL- and RL-specific substrates according to the protocol supplied by the manufacturer. Light emission was measured 2 seconds after addition of each of the substrates and integrated over a 10-second interval. All experiments were performed in duplicates and were repeated at least three times using different DNA preparations.

### *In vitro *transcription

pRF and pBS-pRF +4/+258 plasmids were linearized prior to transcription by *Bam*HI restriction, purified by incubation at 50°C for 30 minutes with 10 μg proteinase K and 0.5% sodium dodecyl sulfate (SDS), and precipitated with 25 mM ethylene diamine tetraacetic acid (EDTA) and 300 mM sodium acetate pH 5.2. Capped RNA transcripts were synthesized by using MAXIscript *in vitro *transcription kit (Ambion) according to the manufacturer's specifications. Briefly, recombinant T7 or T3 polymerases were used to synthesize mRNA from 2.5 μg linearized DNA and 0.5 mM Ribo m^7 ^G Cap Analog (Promega) was added to the reaction mix. *In vitro *transcription was performed by incubation at 37°C for 1 hour in 40 U of RNAsin RNAse inhibitor (Promega). Following transcription, reactions were treated with DNAse I for 15 minutes at 37°C.

### Cell culture, DNA and mRNA transfection and immunofluorescence

HeLa, HEK293 and NSC34 cells were cultured in Dulbecco's Modified Eagle's Media (Euroclone) supplemented with 10% or 5% fetal bovine serum, 200 U/ml penicillin, 200 μg/ml streptomycin and 2 mM glutamine. SH-SY-5Y cells were cultured in Minimum Essential Media (Euroclone) supplemented with 10% Fetalclone III (Hyclone), 200 U/ml penicillin, 200 μg/ml streptomycin and 2 mM glutamine. All cultures were grown as a monolayer in a humidified incubator at 37°C in an atmosphere of 5% CO_2_.

Transient DNA transfections were performed by using Lipofectamine 2000 (Invitrogen) according to the manufacturer's specifications. Briefly, 8 × 10^4 ^cells per well of a 24-well plate were seeded the day prior to transfection. Cells were transfected with DNA (500 ng) and cultured for an additional 24 or 48 hours. In cotransfection experiments, pRL-CMV DNA was added in 1:100 ratio.

mRNA transfections were performed by using Transmessenger Transfection Reagent (Qiagen) according to the manufacturer's specifications. Briefly, 8 × 10^4 ^cells per well of a 24-well plate were seeded the day prior to transfection. Cells were transfected with mRNA (2 μg) and cultured for an additional 8 hours.

Immunofluorescences were performed as described previously [3].

### Sodium dodecyl sulfate-polyacrylamide gel electrophoresis and immunoblotting

Cells were scraped in phosphate-buffered saline and lysed for 30 minutes in RIPA buffer (50 mM Tris-HCl, 1% NP-40, 0.25% Na-deoxycholate, 150 mM NaCl, 1 mM EDTA, pH 7.4) and protease inhibitor cocktail (Sigma-Aldrich) in ice. Protein samples were resuspended in SDS sample buffer and subjected to standard sodium dodecyl sulfate-polyacrylamide gel electrophoresis (SDS-PAGE) followed by protein transfer to a polyvinylidene difluoride membrane (Amersham). Spastin was revealed by immunoblotting with S51 polyclonal antibody [7].

### 5'-end RACE

Total RNA was extracted from cells using TRIzol (Invitrogen) according to the manufacturer's specifications. Rapid amplification of 5'-ends cDNA was carried out using a FirstChoice RLM-RACE kit (Ambion) according to the manufacturer's instructions with the following exceptions. The outer PCR reaction was carried out with 10 pmol gene-specific outer primer (5'-ACCATTCCACAGCTTGCTCCTTCT-3'), 1.25 units of *Pfu *Ultra (Stratagene) and 1.5 ng first-strand cDNA reaction. The PCR conditions were as follows: (1×) 94°C, 3 minutes; (35×) 94°C, 30 seconds; 55°C, 30 seconds; 72°C, 90 seconds; (1×) 72°C, 10 minutes. The inner PCR reaction was carried out with 10 pmol gene-specific inner primer (5'-CGC**AAGCTT**AGGCCTGTTTGTGGAAGACTCGGACG-3', *BamH*I site in bold), using the same conditions as for the outer PCR. PCR products were separated on 2% agarose gel, cloned *BamH*I into a pBluescript KS-vector (Stratagene) and sequenced.

### Bioinformatics analysis

Conservation studies were performed using the human BLAT search database [33]. Alignments between human and mouse sequences were performed by using mVISTA [34] with the following parameters: min_Y (minimum *Y *value on the mVISTA plot) 40%, min_id (minimum conservation identity) 50%, min_length (minimum length for a CNS) 50 bp. IRES prediction was performed by using UTRScan [35]. Transcription factors binding sites were predicted using two different matrixes. MATCH™ [36] parameters: profile, vertebrates; cut-off selection, minimize the sum of both error rates. PATCH™ [36] parameters: sites selection, vertebrate sites; minimum length of site, 10 bp; maximum number of mismatches, 0; mismatch penalty, 100; lower score boundary, 87.5. Bio-informatic analysis of 5'-end full-length *SPG4 *cDNas was performed using the CAGE analysis website [37] and the DBTSS [38].

### Statistical analysis

Data are expressed as the mean ± standard error of the mean. Statistical analysis was performed using a two-way unpaired Student's *t *test.

## List of abbreviations

bp: base pair; DBTSS: database of transcriptional start sites; EDTA: ethylene diamine tetraacetic acid; FL: firefly luciferase; HSP: hereditary spastic paraplegia; IRES: internal ribosome entry site; ORF: open reading frame; PCR: polymerase chain reaction; RL: *Renilla *luciferase; TAP: tobacco acid pyrophosphatase; SDS: sodium dodecyl sulfate; TSS: transcriptional start sites.

## Authors' contributions

GM carried out all of the experiments. EIR conceived of and coordinated the study and wrote the manuscript. Both authors read and approved the final manuscript.

## Supplementary Material

Additional File 1 **Bioinformatic analysis of *SPG4 *transcription start sites based on cap analysis of gene expression**. Position of *SPG4 *tag clusters (TC, red arrows) shown in the CAGE database, in respect to the reference transcripts. The *SPG4 *promoter appears to belong to a broad type that can initiate transcription over a large region resulting in a population of mRNAs with different lengths. Notably, in the case of *SPG4*, these different transcripts may correspond to different coding regions. Indeed the T02F01EB5812 cluster maps between the first and second ATG. It corresponds to 14 mapped tags deriving from different libraries, including nervous tissues. Click here for file

Additional File 2 **Inspection of the *SPG4 *alternative promoter region from the database of transcriptional start sites in HEK293 cells**. Positions of transcriptional start sites are indicated by red lines. The coding sequence is highlighted in yellow. Click here for file
