# Supplementary figures and images for: A cryptic promoter in the first exon of the SPG4 gene directs the synthesis of the 60-kDa spastin isoform
Source: BMC Biol. 2008 Jul 9;6:31. doi: 10.1186/1741-7007-6-31 (PMC2474578; doi:10.1186/1741-7007-6-31)

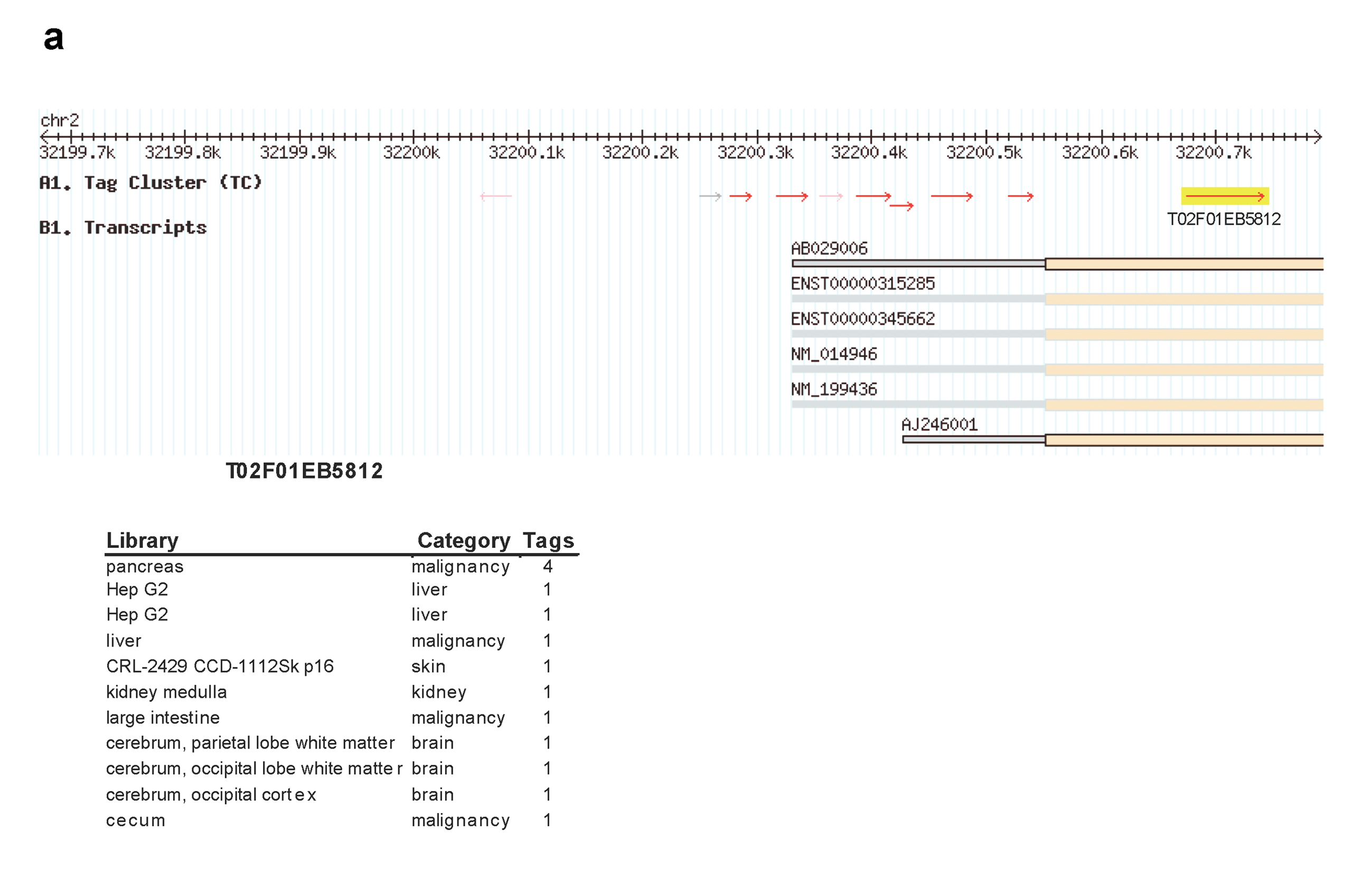

Supplement: Additional File 1 — Bioinformatic analysis of SPG4 transcription start sites based on cap analysis of gene expression. Position of SPG4 tag clusters (TC, red arrows) shown in the CAGE database, in respect to the reference transcripts. The SPG4 promoter appears to belong to a broad type that can initiate transcription over a large region resulting in a population of mRNAs with different lengths. Notably, in the case of SPG4, these different transcripts may correspond to different coding regions. Indeed the T02F01EB5812 cluster maps between the first and second ATG. It corresponds to 14 mapped tags deriving from different libraries, including nervous tissues. [file 1741-7007-6-31-S1.tiff]

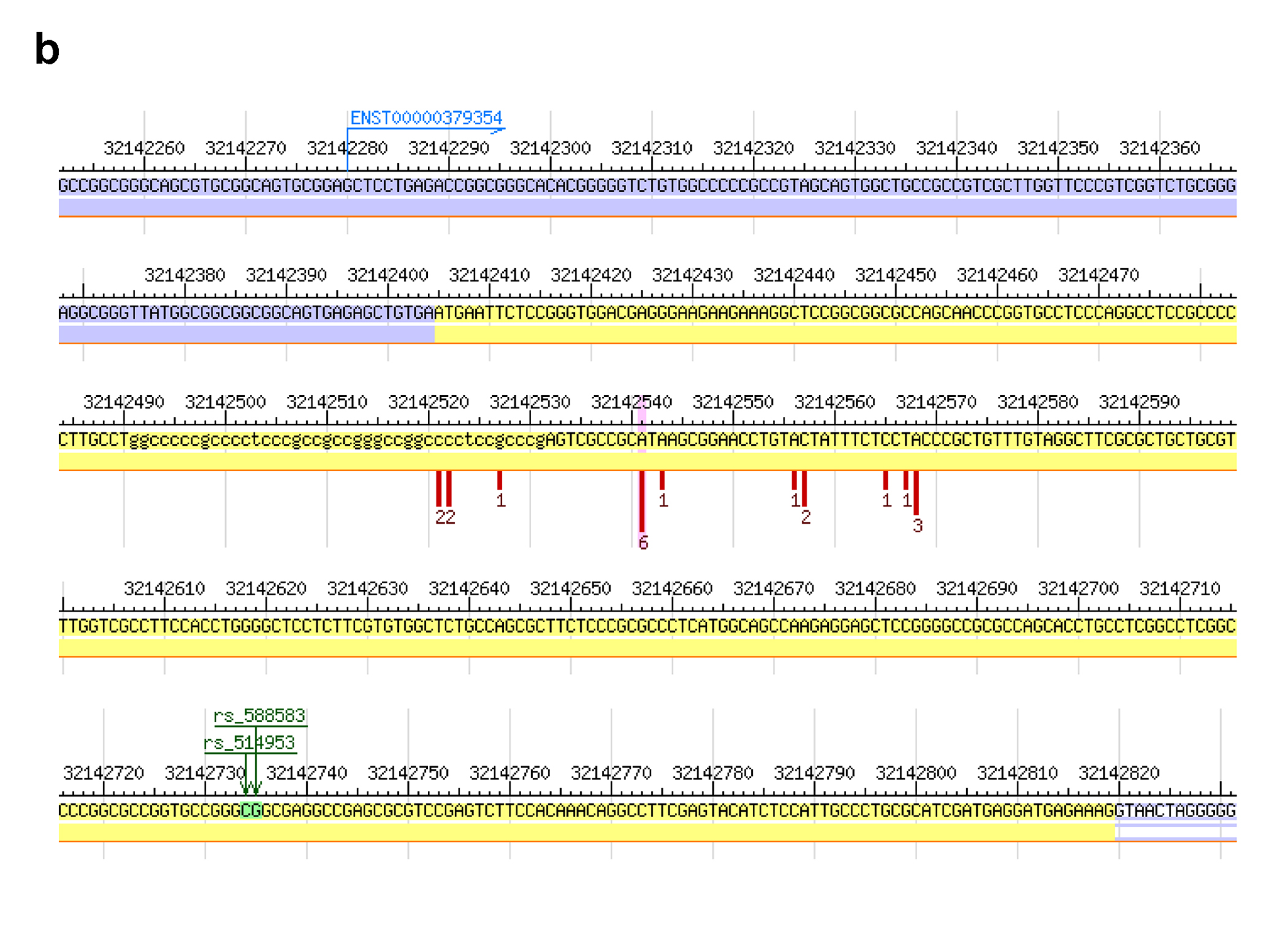

Supplement: Additional File 2 — Inspection of the SPG4 alternative promoter region from the database of transcriptional start sites in HEK293 cells. Positions of transcriptional start sites are indicated by red lines. The coding sequence is highlighted in yellow. [file 1741-7007-6-31-S2.tiff]
